# Differentiating Burnout from Depression: Personality Matters!

**DOI:** 10.3389/fpsyt.2015.00113

**Published:** 2015-08-13

**Authors:** Martin Christoph Melchers, Thomas Plieger, Rolf Meermann, Martin Reuter

**Affiliations:** ^1^Department of Psychology, University of Bonn, Bonn, Germany; ^2^AHG Psychosomatic Hospital, Bad Pyrmont, Germany; ^3^Center for Economics and Neuroscience (CENs), University of Bonn, Bonn, Germany

**Keywords:** TCI, depression, burnout, path analyses, MBI-GS, BDI 2

## Abstract

Stress-related affective disorders have been identified as a core health problem of the twenty-first century. In the endeavor to identify vulnerability factors, personality has been discussed as a major factor explaining and predicting disorders like *depression* or *burnout*. An unsolved question is whether there are specific personality factors allowing differentiation of *burnout* from *depression*. The present study tested the relation between one of the most prominent, biological personality theories, Cloninger’s *Temperament and Character Inventory*, and common measures of *burnout* (*Maslach Burnout Inventory General*) and *depression* (*Beck Depression Inventory 2*) in a sample of German employees (*N* = 944) and a sample of inpatients (*N* = 425). Although the same personality traits (*harm avoidance* and *self-directedness*) were predominantly associated with *burnout* and *depression*, there was a much stronger association to *depression* than to *burnout* in both samples. Besides, we observed specific associations between personality traits and subcomponents of burnout. Our results underline differences in the association of burnout vs. depression to personality, which may mirror differences in scope. While symptoms of *depression* affect all aspects of life, *burnout* is supposed to be specifically related to the workplace and its requirements. The much stronger association of personality to depression can be important to select appropriate therapy methods and to develop a more specified treatment for burnout in comparison to depression.

## Introduction

Work stress has been defined as occurring when the perceived job demands surpass employees’ resources to get their job done [e.g., Ref. (1)]. Among others, symptoms of permanent work stress include physiological consequences like increased risk for cardiovascular diseases [e.g., Ref. (2)], emotional consequences like mood disorders [e.g., Ref. (3)], or intellectual consequences like loss of attention [e.g., Ref. (4)]. Job stress-related affective problems, especially the burnout syndrome, have been a focus of research for years.

Freudenberger (5) and Maslach (6) were the first to investigate the burnout concept, which has [according to the three dimensions of the *Maslach Burnout Inventory General (MBI-GS)*] been defined as consisting of *emotional exhaustion* (job-related symptoms of fatigue), *cynicism* (indifferent or distant attitude toward the job), and reduced *professional efficacy* (individual expectations of continued effectiveness at work) (7). Although by now *burnout* has been identified in virtually all occupational groups, it is still not accepted as an autonomous diagnosis according to DSM or ICD. One of the major reasons for this is the uncertainty whether *burnout* is an independent disorder apart from *depression*. Research in this field has predominantly focused on possible distinctions between both concepts by use of factor analytic approaches (8, 9), by investigating the relationship between *depression* and facets of *burnout* [e.g., Ref. (10–12)], or by considering changes in the relationship of both dependent on progression (13) and severity (14) of the *burnout* syndrome.

An important factor, which has been associated with both *burnout* and *depression*, is personality [e.g., Ref. (15–17)]. For example, relations have been shown to the *Big Five* personality concept [positive correlations especially to *neuroticism*; e.g., Ref. (18, 19)], locus of control [positive correlation to external locus of control; e.g., Ref. (20–22)], or Cloninger’s biosocial model of personality [especially positive correlations with *harm avoidance* and negative correlations with *self-directedness*; e.g., Ref. (23, 24)]. Particularly, employees who display high levels of *harm avoidance*, an external locus of control, passive or defensive coping styles, low levels of hardiness, or poor self-esteem have been identified as vulnerable for *burnout* [compare Ref. (25)].

Based on the previous research and as the distinction between *depression* and *burnout* is still a matter of debate, the current study investigates whether *burnout* and *depression* can be differentiated by their relationship to personality. In this paper, we compare the relationship of *Cloninger’s Temperament and Character Inventory* [*TCI*; (26)] to *burnout (as measured by the MBI)* and to *depression* as measured by the *Beck Depression Inventory 2* [*BDI 2*, (27)] in two independent samples. The first sample consists of 944 German employees, the second sample of 425 patients currently treated in psychosomatic clinics. Our aim is to find out whether the biologically oriented personality theory of Robert Cloninger is suitable to discriminate *burnout* from *depression*, and if putative differences between *burnout* and *depression* can be found in both the sample of healthy participants and the inpatient sample. Considering two independent samples, which are located in different regions of the health-illness continuum allows making statements about whether associations (and putative differences in association) between personality and depression or burnout, respectively, can be generalized.

To our knowledge, this is the first time that the *MBI* and the *BDI 2* have been simultaneously related to Cloninger’s *TCI* in a large not occupationally specific sample. Therefore, a second aim of this paper is to replicate findings on the *TCI/MBI* relationship that have been reported for specific occupational groups [e.g., Ref. (28, 29)], which especially point out the relevance of *harm avoidance* and *self-directedness* for *burnout*.

## Materials and Methods

### Participants

We investigated two samples in this study: the first sample consisted of *N* = 944 German employees [362 male, *M*_(age)_ = 41.0 years; 582 female, *M*_(age)_ = 39.3 years] from a wide range of professions. The second sample included *N* = 425 inpatients [146 male, *M*_(age)_ = 48.0 years; 279 female, *M*_(age)_ = 47.54 years] from psychosomatic clinics. Both samples were recruited for the *Bonn Burnout Research Project (BBRP)*. Written informed consent to participate was obtained prior to the study, which was approved by the local ethics committee at the University of Bonn. Information on participants’ medical and psychiatric background was collected by use of the *SCL-90-R* questionnaire (30).

### Measures

Participants were recruited for a large scale project on the genetic and epigenetic causes of *burnout*. For the healthy sample, recruitment of participants was operationalized by contacting German companies spread over the whole country asking for allowance to invite their employees to take part in our research project and by randomly sent postal invitations to participate in the study to private households in the City of Bonn. Main goal was to include participants of diverse vocational backgrounds to obtain results, which are not restricted to a single profession and its specific characteristics. For the sample of patients, we collaborated with several psychosomatic hospitals spread across Germany, most of them belonging to the AHG consortium. The AHG comprises more than 40 hospitals and has an own research department that supported us in this scientific project. All participants completed a battery of questionnaires with separate sections for demographic information, personality (*TCI*), *depression* (*BDI 2*), and *burnout* (*MBI*). In case of the latter, we used the *MBI-GS* version, which allows measurement of *burnout* in different occupational groups.

The TCI is a seven-dimensional questionnaire consisting of the four temperaments novelty seeking (exploratory activity in response to novel stimulation, impulsive decision making, extravagance in approach to reward cues, quick loss of temper, and avoidance of frustration), harm avoidance (pessimism, shyness, excessive worrying, fearfulness, and easily fatigue), reward dependence (tendency to respond markedly to signals of reward and learning to maintain and pursue behaviors, which were previously associated with such reward), and persistence (perseverance in spite of fatigue or frustration) and the three character dimensions self-directedness (the perceived ability to regulate and adapt behavior to the demands of a situation in order to achieve personally chosen goals and values), cooperativeness (the degree to which a person is generally agreeable in their relations with other people as opposed to aggressively self-centered and hostile) and self-transcendence (the amount of experienced spiritual ideas) (26). According to Cloninger, the temperament dimensions are highly heritable and are based on the activity of the dopaminergic, serotonergic, and noradrenergic neurotransmitter system, while the character dimensions are closely related to environmental influences.

The MBI-GS is a measure for burnout in all professions. It consists of the three subscales: exhaustion (feelings of fatigue), cynicism (indifferent or distanced attitudes toward work), and professional efficacy (expectations of continued effectiveness at work). All of its questions ask specifically for experiences made in a work environment (e.g., “I feel used up at the end of a workday”; “In my opinion I am good at my job”).

### Statistical analysis

**Figure 1:**
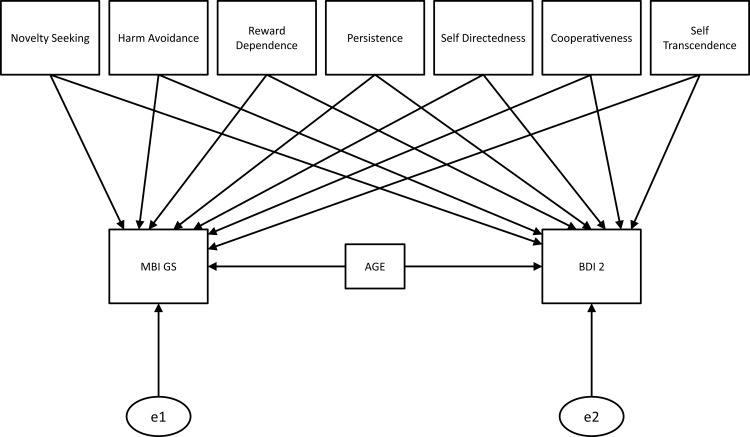
**Path model without restrictions**. Intercorrelation is expected between all seven dimensions of the TCI. All dimensions and age are used to predict participants values in MBI scores (sum score, and scores for exhaustion, efficacy, and cynicism) and BDI 2 scores. Next to this unrestricted model, models, which assume the same regression weights between a TCI dimension and burnout/depression, were tested. Finally, a model assuming the same regression weights for all TCI-scales was tested.

Initially, descriptive statistics and internal consistencies were calculated for all measures. Furthermore, the data of both samples were compared for significant differences. The relationships between the seven dimensions of the *TCI* and the *MBI-GS* or the *BDI 2*, respectively, were analyzed by means of correlations. Age was added as a control variable, and correlation coefficients were calculated separately for female and male participants and tested for significant differences. Furthermore, path models were calculated with the *TCI* subscales as exogenous variables and the *MBI* scores (we used a sum score as well as the three subscale scores) and the *BDI 2* sum score as endogenous variables. Next to an unrestricted model, we tested restricted models assuming the same regression weights (path coefficients) for the relationship of a personality dimension to *burnout* and *depression* (e.g., *harm avoidance* → *MBI* = *harm avoidance* → *BDI 2*). This was done for each of the seven *TCI* dimensions (only one at a time) in order to test whether solutions with the same weights of personality dimensions for *burnout* and *depression* fit significantly worse, which would imply differences in the importance of the respective subscale for *burnout* vs. *depression*. Finally, a model comprising all individual constraints described above was tested (*harm avoidance* → *MBI* = *harm avoidance* → *BDI 2 *+ *novelty seeking* → *MBI* = *novelty seeking* → *BDI 2*…) in order to test a model, which does not assume any differences in the relation of TCI dimensions to *burnout* vs. *depression*. As this model showed a significantly poorer fit for all comparisons in both samples, it is not considered further in depth. Models were fitted using the structural equation modeling software package AMOS (31). Compare Figure 1.

## Results

### Questionnaires and control variables

Means, SDs, sample comparison, and Cronbach’s alpha of participants’ questionnaire responses are depicted in Table 1. Our results fit nicely with normative data on responses to the *TCI* (32), the *BDI 2* (33), and the *MBI* (34) in German samples.

**Table 1:** **Means and SDs of employed participants’ (*N* = 944) and patients’ (*N* = 425) questionnaire responses plus reliability (coefficients in terms of Cronbach’s alpha) of the used scales are presented**.

	*M* (SD) employees	*M* (SD) patients	Cronbach’s alpha (employees/patients)	Number of items	Comparison employees/patients
BDI 2	9.07 (8.04)	23.14 (11.02)	0.91/0.92	21	*F*_(1, 1357)_ = 666.243, *p* < 0.001
MBI exhaustion	2.18 (1.31)	4.63 (1.25)	0.88/0.87	5	*F*_(1, 1357)_ = 972.756, *p* < 0.001
MBI efficacy^a^	1.25 (0.97)	1.82 (1.26)	0.81/0.82	6	*F*_(1, 1357)_ = 95.422, *p* < 0.001
MBI cynicism	1.63 (1.35)	3.21 (1.55)	0.84/0.81	5	*F*_(1, 1357)_ = 347.606, *p* < 0.001
MBI sum score	1.66 (0.93)	3.14 (0.99)	0.86/0.88	16	*F*_(1, 1357)_ = 685.491, *p* < 0.001
TCI NS	20.49 (5.78)	17.08 (6.06)	0.76/0.74	40	*F*_(1, 1357)_ = 79.941, *p* < 0.001
TCI HA	15.03 (6.87)	22.68 (6.77)	0.86/0.83	35	*F*_(1, 1357)_ = 380.521, *p* < 0.001
TCI RD	15.69 (3.80)	15.91 (3.69)	0.71/0.72	24	n.s.
TCI PER	4.50 (1.95)	5.16 (1.93)	0.62/0.61	8	*F*_(1, 1357)_ = 37.068, *p* < 0.001
TCI SD	32.73 (7.08)	26.41 (8.29)	0.86/0.89	44	*F*_(1, 1357)_ = 242.654, *p* < 0.001
TCI C	33.05 (5.01)	31.98 (5.70)	0.76/0.78	42	*F*_(1, 1357)_ = 15.505, *p* < 0.001
TCI ST	10.90 (6.00)	11.12 (5.51)	0.85/0.81	33	n.s.

*^a^Scores on MBI efficacy have been inverted to allow easier interpretation of the scales meaning for general burnout*.

After correction for age, comparison of the questionnaire responses of both samples delivered significant results for all measures except *reward dependence* and *self-transcendence*.

### Relation of burnout, depression, and TCI

Table 2 shows the correlations between all questionnaire measures for the healthy and the inpatient sample.

**Table 2:** **Correlations of TCI, BDI 2, and MBI-GS**.

	TCI NS	TCI HA	TCI RD	TCI P	TCI SD	TCI C	TCI ST	BDI 2	MBI exhaustion	MBI professional efficacy^a^	MBI cynicism	MBI total score
TCI NS	x	**−0.385^###^**	**0.144^###^**	**−0.191^###^**	n.s.	n.s.	**0.152^##^**	**−0.111^#^**	**−**0.091**	**−**0.075*	**−**0.082*	**−**0.106**
TCI HA	**−0.441^###^**	x	0.092**	**−**0.102**	**−0.542^###^**	**−0.198^###^**	n.s.	**0.547^###^**	**0.424^###^**	**0.310^###^**	**0.364^###^**	**0.474^###^**
TCI RD	n.s.	n.s.	x	n.s.	n.s.	**0.417^###^**	**0.253^###^**	n.s.	n.s.	n.s.	**−**0.080*	n.s.
TCI P	**−0.211^###^**	n.s.	n.s.	x	n.s.	n.s.	**0.123^#^**	n.s.	0.093**	**−**0.100**	n.s.	n.s.
TCI SD	0.150*	**−0.586^###^**	n.s.	0.107*	x	**0.359^###^**	**−0.110^#^**	**−0.665^###^**	**−0.430^###^**	**−0.363^###^**	**−0.481^###^**	**−0.551^###^**
TCI C	n.s.	**−**0.149*	**0.422^###^**	0.112*	**0.339^###^**	x	0.083*	**−0.257^###^**	**−0.187^###^**	**−0.131^##^**	**−0.283^###^**	**−0.262^###^**
TCI ST	**0.225^###^**	**−0.173^#^**	**0.199^##^**	n.s.	n.s.	0.109*	x	**0.150^###^**	**0.136^##^**	**−**0.071*	n.s.	n.s.
BDI 2	**−0.180^#^**	**0.516^###^**	n.s.	n.s.	**−0.599^###^**	**−**0.113*	n.s.	x	**0.591^###^**	**0.325^###^**	**0.525^###^**	**0.628^###^**
MBI exhaustion	**−**0.127**	**0.315^###^**	n.s.	0.101*	**−0.277^###^**	**−**0.125*	n.s.	**0.359^###^**	x	**0.214^###^**	**0.609^###^**	**0.803^###^**
MBI prof. efficacy^a^	**−0.213^###^**	**0.285^###^**	n.s.	**−**0.113*	**−0.343^###^**	n.s.	**−0.206^###^**	**0.333^###^**	**0.173^#^**	x	**0.259^###^**	**0.649^###^**
MBI cynicism	n.s.	**0.185^##^**	**−0.222^###^**	n.s.	**−0.342^###^**	**−0.277^###^**	n.s.	**0.284^###^**	**0.448^###^**	**0.304^###^**	x	**0.861^###^**
MBI total	**−0.177^#^**	**0.350^###^**	**−0.180^#^**	n.s.	**−0.435**^###^	**−0.214**^###^	**−**0.128**	**0.441^###^**	**0.700^###^**	**0.689^###^**	**0.809^###^**	x

*^a^Scores on MBI efficacy have been inverted to allow easier interpretation of the scales meaning for general burnout*.

*p ≤ 0.05; **p ≤ 0.01; ***p ≤ 0.001; ^#^*p* ≤ 0.00076; ^##^*p* ≤ 0.00015; ^###^*p* ≤ 0.000015 (adjusted for multiple comparison).

*Coefficients with gray shading describe numbers for the patient sample, numbers without shading coefficients for the employees. Due to multiple testing (66 comparisons), the alpha-levels for significant results were adjusted to *p* = 0.00076, *p* = 0.00015, and *p* = 0.000015 according to Bonferroni correction. Significant results after correction are printed bold. Results have been corrected for age. All coefficients were calculated separately for the female and the male subsample. Results showed no significant differences depending on gender*.

*Harm avoidance* exhibited a medium to large size positive correlation to burnout as well as to depression. *Self-directedness* and (to a lesser extent in case of the patients) *cooperativeness* were negatively related to burnout and depression. *Novelty seeking*, *reward dependence*, *persistence*, and *self-transcendence* revealed very small correlations to both constructs, which in most cases were not significant after Bonferroni correction. Depression and burnout in turn displayed medium to large size correlations (*professional efficacy* exhibited a much smaller relation to depression than the other two subscales of the *MBI-GS* in case of the employee sample). Most interestingly, relations between burnout and depression were much smaller in the patient sample compared to the employee sample. There were no significant differences in size of correlations depending on gender for any measure in both samples.

### Path model analyses

Table 3 represents the path estimations of the path model for *BDI 2* and the scores of the *MBI-GS* (sum score, *exhaustion*, *professional efficacy*, and *cynicism*).

**Table 3:** **Estimated standardized regression weights between the seven TCI dimensions and burnout/depression in the unrestricted model**.

	Novelty seeking	Harm avoidance	Reward dependence	Persistence	Self- directedness	Cooperativeness	Self- transcendence	Expl. of variance (%)
BDI 2	0.055; *p* = 0.039	0.324; *p* < 0.001	−0.086; *p* = 0.001	0.104; *p* < 0.001	−0.488; *p* < 0.001	n.s.	0.102; *p* < 0.001	37.3
BDI 2 (patients)	n.s.	0.288; *p* < 0.001	n.s.	0.146; *p* < 0.001	−0.501; *p* < 0.001	0.104; *p* = 0.015	n.s.	36.6
MBI sum score	n.s.	0.279; *p* < 0.001	n.s.	n.s.	−0.387; *p* < 0.001	n.s.	n.s.	22.8
MBI sum score (patients)	n.s.	0.137; *p* = 0.022	−0.171; *p* < 0.001	n.s.	−0.352; *p* < 0.001	n.s.	n.s.	17.2
MBI exhaustion	0.068; *p* = 0.038	0.335; *p* < 0.001	n.s.	0.140; *p* < 0.001	−0.234; *p* < 0.001	n.s.	0.100; *p* < 0.001	19.6
MBI exhaustion (patients)	n.s.	0.284; *p* < 0.001	−0.110; *p* = 0.033	0.137; *p* = 0.003	−0.132; *p* = 0.027	n.s.	n.s.	12.9
MBI professional efficacy	n.s.	0.151; *p* < 0.001	n.s.	n.s.	−0.302; *p* < 0.001	n.s.	−0.090; *p* = 0.004	12.2
MBI professional efficacy (patients)	−0.136; *p* = 0.008	n.s.	n.s.	−0.104; *p* = 0.022	−0.333; *p* < 0.001	0.112; *p* = 0.031	−0.135; *p* = 0.004	17.2
MBI cynicism	n.s.	0.158; *p* < 0.001	n.s.	n.s.	−0.363; *p* < 0.001	−0.096; *p* = 0.004	n.s.	17.2
MBI cynicism (patients)	n.s.	n.s.	−0.170; *p* < 0.001	n.s.	−0.307; *p* < 0.001	n.s.	n.s.	12.3

*Age was added as an additional exogenous variable*.

With exception of *exhaustion* in case of the employee sample, low *self-directedness* turned out to be the by far most important path with up to 25.1% (*BDI 2*) of explained variance (squared path coefficient). The second most important predictor was high *harm avoidance* with up to 11.2% explained variance (*MBI exhaustion*). Overall, the *TCI* was able to explain much more variance in depression than in burnout.

Results of the model comparison between the restricted and the unrestricted models (for *BDI 2* vs. *MBI sum* score) are shown in Tables 4 and 5. Here, we stepwise set all paths between a *TCI* dimension and burnout/depression invariant one after another (but only one at a time). Results show that, especially in case of *self-directedness*, but also in case of *harm avoidance*, *persistence*, and *self-transcendence*, the models assuming differences in paths to burnout and depression fit significantly better to the data than the restricted models (equality constraints), which means we can assume that in these cases the relationship of the respective *TCI* scale to burnout is significantly smaller than the one to depression. For *reward dependence*, the difference between depression and burnout is only significant for the employee sample, while in case of *cooperativeness* the same is true for the patient sample. We performed the same model comparisons for *depression* vs. the *MBI* subscales (e.g., *BDI 2* vs. *exhaustion*) as well as within the *MBI* subscales (e.g., *exhaustion* vs. *cynicism*). The summarized results are presented in Tables 6 and 7.

**Table 4:** **Results of the path analyses comparing MBI sum score and BDI 2 for the employee sample**.

Model	CMIN	X^2^/df	df model	*p* model	CFI	Difference test X^2^ (unrestricted model)
Unrestricted model	201.984	25.248	8	<0.001	0.918	–
Novelty seeking same for BDI and MBI	205.522	22.836	9	<0.001	0.917	n.s.
Harm avoidance same for BDI and MBI	285.203	31.689	9	<0.001	0.883	X^2^ = 83.219; *p* < 0.001
Reward dependence same for BDI and MBI	210.461	23.385	9	<0.001	0.915	X^2^ = 8.477; *p* = 0.004
Persistence same for BDI and MBI	219.060	24.340	9	<0.001	0.911	X^2^ = 17.076; *p* < 0.001
Self-directedness same for BDI and MBI	404.068	44.896	9	<0.001	0.833	X^2^ = 202.084; *p* < 0.001
Cooperativeness same for BDI and MBI	202.046	22.450	9	<0.001	0.918	n.s.
Self-transcendence same for BDI and MBI	218.567	24.285	9	<0.001	0.911	X^2^ = 16.583; *p* < 0.001
All subscales same for BDI and MBI	791.272	52.751	15	<0.001	0.672	X^2^ = 589.288; *p* < 0.001

*The table shows the fit of a liberal model (unrestricted model) compared to nested models, which assume the same regression weights between an individual TCI dimension and burnout/depression, while all other respective dimensions stay free (compare Materials and Methods). Furthermore, a model, which assumes the same regression weights for all dimensions, is tested*.

**Table 5:** **Results of the path analyses comparing MBI sum score and BDI 2 for the patient sample**.

Model	CMIN	X^2^/df	df model	*p* model	CFI	Difference test X^2^ (unrestricted model)
Unrestricted model	80.560	10.070	8	<0.001	0.923	–
Novelty seeking same for BDI and MBI	81.608	9.068	9	<0.001	0.923	n.s.
Harm avoidance same for BDI and MBI	108.651	12.072	9	<0.001	0.894	X^2^ = 28.091; *p* < 0.001
Reward dependence same for BDI and MBI	82.805	9.201	9	<0.001	0.992	n.s.
Persistence same for BDI and MBI	95.210	10.579	9	<0.001	0.909	X^2^ = 14.650; *p* < 0.001
Self-directedness same for BDI and MBI	162.861	18.096	9	<0.001	0.837	X^2^ = 82.301; *p* < 0.001
Cooperativeness same for BDI and MBI	86.386	9.598	9	<0.001	0.918	X^2^ = 5.826; *p* = 0.016
Self-transcendence same for BDI and MBI	84.428	9.381	9	<0.001	0.92	X^2^ = 3.867; *p* = 0.049
All subscales same for BDI and MBI	302.394	20.160	15	<0.001	0.213	X^2^ = 221.833; *p* < 0.001

*The table shows the fit of a liberal model (unrestricted model) compared to nested models, which assume the same regression weights between an individual TCI dimension and burnout/depression, while all other respective dimensions stay free (compare Materials and Methods). Furthermore, a model, which assumes the same regression weights for all dimensions, is tested*.

**Table 6:** **Results of the path analyses comparing MBI subscale scores with each other and with the BDI 2 (employee sample)**.

	Novelty seeking same	Harm avoidance same	Reward dependence same	Persistence same	Self- Directedness same	Cooperativeness same	Self- Transcendence same
BDI 2 vs. MBI exhaustion	n.s.	X^2^ = 70.012; *p* < 0.001	X^2^ = 8.578; *p* = 0.003	X^2^ = 11.060; *p* = 0.001	X^2^ = 204.267; *p* < 0.001	n.s.	X^2^ = 11.840; *p* = 0.001
BDI 2 vs. MBI professional efficacy	X^2^ = 4.059; *p* = 0.044	X^2^ = 90.453; *p* < 0.001	X^2^ = 8.946; *p* = 0.003	X^2^ = 21.081; *p* < 0.001	X^2^ = 207.848; *p* < 0.001	n.s.	X^2^ = 20.750; *p* < 0.001
BDI 2 vs. MBI cynicism	n.s.	X^2^ = 84.600; *p* < 0.001	X^2^ = 7.500; *p* = 0.006	X^2^ = 18.353; *p* < 0.001	X^2^ = 185.644; *p* < 0.001	n.s.	X^2^ = 16.341; *p* < 0.001
MBI exhaustion vs. MBI professional efficacy	n.s.	X^2^ = 20.833; *p* < 0.001	n.s.	X^2^ = 24.566; *p* < 0.001	n.s.	n.s.	X^2^ = 18.949; *p* < 0.001
MBI exhaustion vs. MBI cynicism	n.s.	X^2^ = 9.898; *p* = 0.002	n.s.	X^2^ = 11.854; *p* = 0.001	X^2^ = 7.165; *p* = 0.007	n.s.	X^2^ = 4.698; *p* = 0.030
MBI professional efficacy vs. MBI cynicism	n.s.	n.s.	n.s.	n.s.	X^2^ = 10.071; *p* = 0.002	X^2^ = 7.933; *p* = 0.005	n.s.

*Once again, an unrestricted model was compared to nested models (compare Tables 4 and 5). Here, only X^2^ values and *p*-values (X^2^ difference test) for the respective path comparison are presented*.

**Table 7:** **Results of the path analyses comparing MBI subscale scores with each other and with the BDI 2 (patient sample)**.

	Novelty seeking same	Harm avoidance same	Reward dependence same	Persistence same	Self- directedness same	Cooperativeness same	Self- transcendence same
BDI 2 vs. MBI exhaustion	n.s.	X^2^ = 24.093; *p* < 0.001	n.s.	X^2^ = 11.609; *p* = 0.001	X^2^ = 87.025; *p* < 0.001	X^2^ = 5.945; *p* = 0.015	n.s.
BDI 2 vs. MBI professional efficacy	n.s.	X^2^ = 29.575; *p* < 0.001	n.s.	X^2^ = 16.857; *p* < 0.001	X^2^ = 79.893; *p* < 0.001	X^2^ = 4.450; *p* = 0.035	X^2^ = 5.128; *p* = 0.024
BDI 2 vs. MBI cynicism	n.s.	X^2^ = 29.813; *p* < 0.001	n.s.	X^2^ = 14.957; *p* < 0.001	X^2^ = 77.653; *p* < 0.001	X^2^ = 7.329; *p* = 0.007	n.s.
MBI exhaustion vs. MBI professional efficacy	X^2^ = 5.365; *p* = 0.021	X^2^ = 7.944; *p* = 0.005	n.s.	X^2^ = 13.247; *p* < 0.001	X^2^ = 5.406; *p* = 0.020	n.s.	X^2^ = 8.536; *p* = 0.003
MBI exhaustion vs. MBI cynicism	n.s.	X^2^ = 7.435; *p* = 0.006	n.s.	X^2^ = 4.906; *p* = 0.027	X^2^ = 6.438; *p* = 0.011	n.s.	n.s.
MBI professional efficacy vs. MBI cynicism	n.s.	n.s.	n.s.	n.s.	n.s.	X^2^ = 8.023; *p* = 0.005	n.s.

*Once again, an unrestricted model was compared to nested models (compare Tables 4 and 5). Here, only X^2^ values and *p*-values (X^2^ difference test) for the respective path comparison are presented*.

The analyses once again emphasize the bigger relevance of *self-directedness* and *harm avoidance* for *depression* compared to the three subscales of the *MBI*. The only exceptions to this are the *BDI 2* vs. *MBI exhaustion* comparisons in case of *harm avoidance* and *persistence* (employee sample) as well as the *BDI 2* vs. *MBI professional efficacy* comparison in case of *self-transcendence* (patient sample). The internal comparison of the subscales of the *MBI* provides fewer significant results concerning differences in relation to the *TCI*. Most prominent in this case is the difference in relation between *persistence* and *exhaustion* or *professional efficacy*, respectively. Here, *persistence* seems especially relevant for prediction of *MBI exhaustion*.

## Discussion

The present study investigated the relationship of Cloninger’s biosocial model of personality (*TCI*) to burnout (*MBI*) and depression (*BDI 2*) in a sample of German employees from various professions and in an independent sample of patients from psychosomatic hospitals. Based on evidence from preceding studies, we expected predominance of the *TCI’s* subscales *harm avoidance* and *self-directedness* to predict participants’ self-reported levels of *burnout* and *depression*. Furthermore, we searched for putative differences in the relationship between personality and *depression* vs. personality and *burnout*, which we wanted to replicate in two samples of participants with varying degrees of emotional stress.

The descriptive data (Table 1) shows that the employees’ responses to the *TCI* overall match normative data for the *TCI*. If we compare our data to the score categorizations (severity of burnout/depression), which have been suggested for the *BDI 2* [compare Ref. (27)] and the *MBI-GS* [compare Ref. (7)], it is evident that our control sample on average exhibits an exposure to depression and burnout. The mean *BDI 2* score would be classified as a “minimal depression.” *MBI exhaustion* and *cynicism* scores would be classified as “moderate exposure to burnout” or middle third in range of experienced burnout. Notably, these results are primarily driven by a minority of employees with very high scores in depression (2.2% with severe depression and 7.7% with a medium depression) or burnout (17–20% high exposure to burnout on all three subscales). This rather high number of active employees with clinical depression or strong exposure to burnout should be considered in future research and in the development and implementation of prevention programs concerned with affective disorders. As expected, we see a much higher exposure to *depression* and *burnout* in the patient sample compared to the employees. Here, the mean *BDI 2* score would be classified as a moderate depression, while scores concerning the *MBI* would be classified as high exposure in case of *exhaustion* and *cynicism* and as moderate exposure in case of *professional efficacy*.

Results of the correlations and path models fit nicely with our expectations: more than one-third of the *BDI 2*’s variance can be explained by the *TCI’s* subscales in both samples, with *self-directedness* and *harm avoidance* as the by far most important predictors. Prediction of the *MBI* subscales reveals a similar pattern for the employee sample: once again, *self-directedness* and *harm avoidance* are the most important factors to explain the subscales. All other dimensions of the *TCI* only play a subordinate role in explaining *MBI* values. Therefore, our study confirms findings from previous studies, which found strong relations between *self-directedness/harm avoidance* and *burnout* in specific (healthy) occupational groups. Out of a personality theory perspective, this result is quite interesting because harm avoidance is a temperament dimension, which according to Cloninger et al. (26) originates mainly from genetic–biological causes while self-directedness is a character dimension, which originates primarily from environmental influences, indicating a gene–environment interaction in the association between personality and depression/burnout. Furthermore, although harm avoidance shares some aspects of its definition with depression and to a lesser extend with burnout, overall self-directedness proved to be the more important predictor. In case of the patient sample, results are a little bit more mixed up concerning prediction of the *MBI* subscales. Here, *persistence* and reward *dependence* play a more important role, while the importance of *harm avoidance* decreases.

A crucial difference between *MBI* and *BDI 2* is the overall amount of variance explained by the respective model: for *exhaustion*, the *TCI* explains 19.6%/12.9% of variance, for *MBI cynicism* 17.2%/12.3%, and for *MBI* professional *efficacy* 12.2%/17.2%. Even for the total burnout score, the amount of explained variance (22.8%/17.2%) does not reach values of the *BDI 2* (37.3%/36.6%). Obviously, prediction of participants’ burnout-scores is considerably less efficient than prediction of scores in *depression*, which means that there is a closer relation of the *TCI* personality concept to *depression* than to *burnout*. A possible explanation for this finding could be differences in the controllability of workplace environment conditions vs. private environment conditions: workplace stress (which is supposed to be the major stressor in burnout) differs from personal or environmental stress (which is a major stressor in *depression*) in such a way that workplace environment offers fewer opportunities to influence framework conditions than personal environment because conditions in the workplace are more predefined than those in private life (14). Individual personality characteristics can have positive or negative effects on the risk for developing an affective disorder, because they influence adaptive or maladaptive behaviors and also internal coping strategies [e.g., Ref. (35, 36)]. This influence can be much more effective if the particular environment offers enough degrees of freedom (for behavior, choices, etc.) to alter the conducive or obstructive environmental conditions by use of personality-specific (adaptive or maladaptive) behavior. Therefore, the fewer degrees of freedom in case of *burnout* relevant environment compared to *depression* relevant environment can explain the differences in the strength of association of personality with *burnout* vs. *depression* that we found in our study. A second argument concerns potential overlapping genetic influences. It has been shown that *depression* [e.g., Ref. (37)] as well as personality [e.g., Ref. (38)] is to a substantial amount influenced by genetic makeup. Although there is first evidence that genetic influences are also relevant for *burnout* [e.g., Ref. (39)], estimations for their effect are significantly lower than in case of *depression* or personality. Therefore, a second explanation for our findings could be the bigger (and potentially shared) amount of genetic influences for personality and *depression*.

The differences in explained variance between the subscales of the *MBI* might reflect differences between the MBI subscales, which have been discussed before in the literature. For example, the positive phrasing of *professional efficacy* as opposed to the negative phrasing of the other two subscales has been discussed as a cause for differences, because direction of phrasing influences response patterns in the *MBI* (40). Furthermore, alternative *MBI-GS* models with an inefficacy scale instead of an efficacy scale exhibit an improved factor structure and higher correlations to the other two subscales (41, 42). A distinction between *professional efficacy* and the other two subscales of the *MBI* is also suggested by results concerning the *MBI-GS’s* factor structure [e.g., Ref. (43–45)].

The comparison of the different prediction models for *depression* and *burnout* (compare Tables 4 and 5 and Tables 6 and 7) yields interesting results concerning the relevance of the *TCI* subscales. First of all, results demonstrate that neither *novelty seeking* nor *cooperativeness* play a noteworthy role for the prediction of *depression* and *burnout*. *Self-directedness* on the other hand is the best predictor for both *depression* and the *MBI* sum score, but at the same time, its association with the subscales of *burnout* is rather uneven. For example, *self-directedness* displays a stronger association to *cynicism* and professional efficacy than to *exhaustion*. This also applies to *harm avoidance* (stronger association to *exhaustion* than to professional *efficacy* and *cynicism*) indicating that the strength of association with both scales might help to distinguish *depression* from *burnout*. Finally, the results concerning *persistence* and *reward dependence* are interesting. Here, *persistence* seems especially related to *MBI exhaustion* but not to the other two subscales of the *MBI*, which makes perfectly sense as high persistence implies high performance over a long time, which may lead to exhaustion. *Reward dependence* on the other hand seems to play a role for prediction of *burnout* in patients, but not in the employee sample. Overall, the latter differences, however, are not of great importance in proportion to the predominating effects of *self-directedness* and *harm avoidance*.

In summary, the following picture emerges: *self-directedness* and *harm avoidance* are the predominantly associated *TCI* dimensions for *depression* as well as for *burnout*, and this seems true for occupation homogenous samples as well as for groups of mixed occupations. In case of patients, the meaning of *harm avoidance* seems to be less important. *Depression* has a much closer relationship to the *TCI* personality concept than *burnout*. This difference is once again mainly driven by *self-directedness* and *harm avoidance*. There is no personality dimension that shows a strong specific association with either *burnout* or *depression* exclusively. Finally, *persistence* and in case of the patient sample *reward dependence* seem suited to distinguish between subscales of the *MBI*.

Our results may have an important impact on choosing the best therapy for depressive vs. burned-out patients. For example, in psychotherapy, personality characteristics have been found to be relevant for interpersonal processes [e.g., Ref. (46)] or learned helplessness [e.g., Ref. (47)]. If *depression* is much closer related to personality than *burnout*, consideration of personality-related therapy blocks (like training of positive self-efficacy expectancy for patients with low *self-directedness* or training to reduce rumination in patients with high *harm avoidance*) may be important in case of depressive disorders. In case of *burnout*, other methods (like training of physical stress relief) might fit better to reach the optimal therapeutic success. In order to develop therapy methods, which are adjusted to the specific symptoms of depression and burnout, future studies should also focus on potential biological factors, which differentiate between both diseases. This approach of disentangling psychological concepts by differences in their biological bases has been applied very successful in other areas of psychiatry [compare, for example, Ref. (48)]. Potential areas of study could, for example, be genetic or epigenetic differences, functional differences, differences in transmitter or hormone activity, or structural brain differences.

We have to point out that our study has some limitations. First of all, although we checked for demographic characteristics, we cannot exclude the possibility that our sample includes a selection bias. It is possible that employees with specific characteristics (e.g., specific opinion on or experiences with *burnout*) are more willing to participate in a study on *burnout* than others. Furthermore, our data are cross-sectional. A longitudinal design would allow checking for causal pathways. Especially, predictions on first-time employees’ psychic health development and proneness to *burnout* and *depression* depending on personality would be of interest. Our current cross-sectional approach involves the risk of underestimating the influence of personality, as *burnout* develops gradually throughout working life and younger employees with *burnout-* or *depression*-prone personality characteristics might not yet be affected. Besides, although personality is defined as rather stable over the life span, research has shown that psychiatric diseases have a tremendous effect on personality self-reports. It can be assumed that the severity of illness is reflected in the extremeness of responses on personality scales in our data. Due to the cross-sectional character of our study, we cannot disentangle causal effects of depression/burnout on personality or *vice versa*. However, many researchers and clinicians are of the opinion that burnout is a prodromal syndrome of depression, i.e., the strength of the association between personality and burnout/depression can reflect such a process model. A related aspect concerns potential prior depressive or burnout episodes, which could also influence questionnaire responses. For future studies, it could be useful to assess number and duration of these prior episodes to control for their putative influence on questionnaire responses. Finally, it could be useful to replicate and validate the results that we found in our study by alternative assessment tools that do not rely on self-report data because self-report data may be prone to some psychometric disadvantages. Nevertheless, self-report measures have indeed their justification and are therefore applied in countless studies and in clinical settings. Especially, the BDI 2 has become a sort of golden standard in the diagnosis of depression. Moreover, several studies have demonstrated high convergent validity between the BDI 2 and standardized psychiatric interviews [for example, to the SCID-I, *r* = 0.083; e.g., Ref. (49, 50)].

## Author Contributions

All authors agreed on the study design and helped collecting and organizing the data for this study. Separate drafts for parts of the manuscript were written by all authors. All authors read and corrected the manuscript. MM managed the literature searches, performed the statistical analyses, and wrote the final manuscript. All authors contributed to and have approved to the final manuscript.

## Conflict of Interest Statement

The authors declare that the research was conducted in the absence of any commercial or financial relationships that could be construed as a potential conflict of interest.
